# Smartphone Use and Social Activities Among People With Mild to Moderate Dementia: Multi-Informant Cross-Sectional Study

**DOI:** 10.2196/81927

**Published:** 2026-04-24

**Authors:** Shuji Tsuda, Satoko Hotta, Hiroshige Matsumoto, Kae Ito

**Affiliations:** 1 Research Team for Human Care, Tokyo Metropolitan Institute for Geriatrics and Gerontology, 35-2 Sakaecho, Tokyo, 173-0015, Japan, 81 339643241; 2 Graduate School of Health Management, Keio University, Kanagawa, Japan; 3 Department of Community Health Nursing/Public Health Nursing, Graduate School of Medicine, The University of Tokyo, Tokyo, Japan

**Keywords:** older adults, dementia, smartphone, everyday information and communication technology, social participation

## Abstract

**Background:**

Smartphones have become deeply embedded in daily life, supporting a range of social and practical activities. Individuals with dementia can potentially use smartphones to compensate for cognitive decline and maintain independence. However, while smartphones are widely studied in controlled research settings, little is known about how individuals with dementia spontaneously use them in everyday life. Understanding usage patterns and their potential link to social participation could inform strategies to support smartphone use in their social and practical daily activities.

**Objective:**

This study aimed to identify factors associated with spontaneous smartphone use, describe usage patterns, and examine whether smartphone use is associated with participation in social activities.

**Methods:**

In 2024, we conducted a cross-sectional survey among community-dwelling individuals with mild to moderate dementia from 17 medical facilities in Tokyo. Structured questionnaires were completed by the participants, their families, and attending physicians. Participation in social activities was assessed using the “Spending Time with Others” subscale of the Social Functioning in Dementia (SF-DEM) scale. Factors associated with smartphone use were analyzed using multinomial logistic regression. Associations with participation in social activities were assessed via hierarchical linear regression.

**Results:**

Among 151 participants with a mean age of 82.9 (SD 6.6) years, 43 (29%) participants were regular smartphone users. Smartphone use was negatively associated with older age (odds ratio [OR] 0.41, 95% CI 0.21‐0.80) and positively associated with longer education (OR 1.82, 95% CI 1.00‐3.30), living alone (OR 3.17, 95% CI 1.08‐9.31), and better cognitive function (OR 2.14, 95% CI 1.24‐3.69). Common uses included calling, texting, taking photos and videos, and checking the news and weather. Smartphone users reported marginally more frequent participation in social activities than nonusers did (b=1.41, 95% CI –0.01 to 2.83), particularly visiting the homes of friends or family (b=0.43, 95% CI 0.08‐0.78) and shopping together (b=0.49, 95% CI 0.15‐0.83).

**Conclusions:**

Despite their limited use at the time of this writing, commercial-based smartphones have the potential to support participation in social activities among individuals with dementia. Targeted support may help bridge the gap between this usage and the broader capabilities of these devices, enhancing their role in sustaining meaningful social participation.

## Introduction

### Background

Given the global trends in population aging and growth, an estimated 57.4 million people were living with dementia in 2019, and this number is projected to increase to 152.8 million by 2050 [1]. Dementia is characterized by progressive cognitive decline that gradually interferes with daily functioning and independence [2]. While subjective experiences vary, many people with dementia can maintain a good quality of life with appropriate support [3-5]. Participation in social activities is a key contributor to their well-being, particularly in the mild to moderate stages [5,6]. However, individuals with dementia tend to engage less in or withdraw from social activities before and after their diagnosis [7]. Although this decline may reflect a general tendency toward disengagement, many individuals still want to continue participating in activities that they find meaningful [8]. Supporting the well-being of people with dementia may therefore involve not only sustaining overall levels of participation but also enabling continued participation in personally meaningful activities [6].

Assistive technology, which ranges from purpose-built devices to research-based apps installed on everyday information and communication technologies (EICTs), has been widely explored as a support tool for people with dementia. It varies from memory aids and task-prompting systems to GPS tracking and communication technologies, targeting domains such as cognitive stimulation, assistance with daily activities, and social participation [9-11]. However, to date, most studies have been small-scale feasibility trials, and their findings often reveal only modest [10-12] or inconclusive effectiveness [13]. While modest evidence supports the use of assistive technology for memory support [10], task prompting [12], safety monitoring [9], and promoting social participation [11], assistive technology is rarely implemented in practice [14]. Users and caregivers frequently report barriers such as limited usability, low awareness, stigma, low accessibility, and inadequate technical support [15]. These challenges highlight the need to explore more pervasive alternatives to support the daily lives of individuals with dementia [10].

EICTs, particularly smartphones, have become deeply embedded in daily life. In 2022, smartphone ownership among adults ranged from 76% to 98% across 18 countries with available data; even among older adults aged 50 years and older, ownership rates reached 55%‐96% [16]. People increasingly rely on smartphones to engage in a variety of social and practical activities, such as sending messages, making video calls, searching for information, making digital payments, navigation, and booking restaurants [17]. The diversity of use reflects the fact that everyday social infrastructure is increasingly designed with smartphone users in mind. These integrated services facilitate self-initiated engagement and support the continuity of personally meaningful routines.

Importantly, some community-dwelling individuals with dementia spontaneously use smartphones in their daily lives [18,19]. They consider smartphones nonstigmatizing and enjoy exploring innovative ways to tailor them to their lifestyles [20]. Additionally, recent studies focused on training interventions have revealed positive learning effects and improved task performance in this population [21,22]. These findings suggest that smartphones can be commonly used by individuals with dementia and may serve as accessible tools to support autonomy and engagement. However, little is known about whether and how people with dementia spontaneously use smartphones and whether they leverage commercially available, but not research-based, apps and functions to maintain participation in social activities [10,11]. Gaining such insights could inform strategies to support individuals with dementia in effectively using commercially available EICTs to participate in their preferred social activities, thereby enhancing their well-being.

### Objectives

In this study, we aimed to explore the role of smartphones in the lives of older adults with mild to moderate dementia. Specifically, we sought to (1) identify factors associated with spontaneous smartphone use, (2) describe these patterns of smartphone use in this population, and (3) examine whether smartphone users engage in more frequent social activities than nonusers do.

## Methods

### Study Design

We conducted a cross-sectional observational survey to explore smartphone use among individuals with mild to moderate dementia. The survey was carried out at 17 medical facilities where certified dementia support doctors, who have completed a 2-day governmental training course on community-based dementia care [23], provide outpatient dementia services. The study was conducted in collaboration with these doctors, who engaged with potential participants through outpatient services and provided their clinical information.

### Study Settings

Between February and August 2024, data were collected from 17 medical facilities in Tokyo, including 4 hospitals and 13 primary care clinics. All participating hospitals were designated medical centers for dementia that provide memory clinic services through their psychiatric departments for outpatients and patients referred from nearby primary care clinics for diagnostic evaluation or management of severe behavioral and psychological symptoms of dementia (BPSD). The 4 hospitals exhibit varied institutional characteristics. They include an acute care hospital, a chronic care hospital, a psychiatric hospital, and a hospital offering a mix of acute and chronic care services, with bed capacities ranging from 126 to 550 beds. This diversity in hospital type and size reflects the heterogeneous nature of medical centers for dementia in Japan and allows inclusion of a range of clinical roles within regional dementia care.

The 13 primary care clinics were generally small, with 1-6 physicians affiliated with each clinic providing outpatient services on a rotating basis. Five clinics with multiple physicians offered dedicated outpatient sessions for dementia care. The remaining clinics provided care for individuals with dementia during general outpatient visits. In both clinic types, certified dementia support doctors typically collaborate with Medical Centers for Dementia to obtain diagnostic consultations and BPSD management. As this collaborative model between medical centers for dementia and primary care clinics is widely implemented throughout Japan under the national dementia policy, our study setting is likely to be representative of outpatient dementia care in other regions [24].

### Participants

At the 17 participating medical facilities, the certified dementia support doctors screened individuals for eligibility based on the following criteria: (1) regularly attended medical appointments at the facility for longer than 6 months, (2) a clinical diagnosis of dementia, (3) a stage of mild cognitive impairment, mild dementia, or moderate dementia as determined using the “Functional Assessment Staging of Alzheimer’s Disease” [25], and (4) sufficient cognitive capacity to provide informed consent. We excluded individuals with advanced medical conditions associated with comorbid conditions that suggested an end-of-life stage. The certified dementia support doctors screened their outpatients on a rolling basis until they reached a predetermined number of participants, which ranged from 5 to 30 depending on the volume of their outpatient clinic.

### Procedure

Data were collected using 2 self-administered questionnaires, one for individuals with dementia (participants) and the other for their family members. Clinical information was recorded on a data sheet by the certified dementia support doctors. The doctors provided the participants and family members with the questionnaires and a return envelope addressed to the first author; the participants were instructed to return the completed questionnaires by postal mail within 2 weeks. The clinical data sheets were returned separately by the doctors via postal mail. A postal reminder was sent to participants if the completed questionnaires were not received within 2 weeks.

Participants were instructed to complete the questionnaires at home, with assistance from their family members only when necessary. To minimize reporting bias, the family members were instructed not to influence the participants’ responses. The family members completed their own questionnaires independently and were requested not to discuss or share their responses with the participants. The doctors were blinded to the participants’ and families’ responses and were instructed to administer the Mini-Mental State Examination (MMSE) based on standardized written guidance.

### Instruments

#### Participant Questionnaire

The participant questionnaire was used to collect the participants’ baseline characteristics, including age, sex, years of education, marital status (married, single, divorced, or widowed), living arrangements (cohabiting or living alone), and perceived economic status (very poor, poor, fair, good, or very good). Factual information such as age and sex was cross-verified using the doctors’ clinical data sheets as references. Sex was confirmed in 148 out of 151 (98%) cases, with the remaining 3 (2%) cases unverified owing to missing responses. For age, 131 out of 151 (87%) entries corresponded, while 17 (11%) entries differed by approximately 1 year, likely owing to differences in the time of data entry or recall errors. The 3 participants who did not report their sex also did not report their age. For consistency, we used the clinical sheet data for these variables.

The participant questionnaire also included a section on their mobile phone use, including daily use (smartphones, cellphones, or no use) and years of use. We provided smartphone users with a list of 25 general smartphone apps and functions (eg, calling, texting, maps, and videos) and inquired whether they used them weekly, occasionally, or never (Multimedia Appendix 1). The selection and categorization of apps and functions were informed by governmental surveys of the general public and by previously published studies examining tablet use among people with dementia [26,27]. Multinomial logistic regression was conducted with nonusers as the reference category. All independent variables were categorized as shown in the parentheses, with the first category used as the reference group in the regression analysis.

#### Family Questionnaire

The family questionnaire was used to collect demographic information, including age, sex, family relationship with the participant, and the frequency of time spent together (categorized as daily, weekly, monthly, or yearly). The remaining sections inquired about the individuals with dementia to assess their daily cognitive functioning, BPSD, and daily social functioning.

To measure daily cognitive functioning, the Dementia Assessment Sheet for Community-based Integrated Care System 8-items (DASC-8) was administered [28]. It is a validated tool for use in community and primary care settings [28] and is widely applied in Japan to assess cognitive and functional decline in older adults. It comprises 8 items covering memory, orientation, and basic and instrumental activities of daily living. Each item was rated by the family member on a scale ranging from 0 (independent) to 3 (fully dependent). In the present study, DASC-8 results demonstrated good internal consistency (Cronbach α=0.83).

To measure BPSD, the 5-item short-form version of the Dementia Behavior Disturbance scale (DBD-5) was used [29]. The DBD-5 is a brief caregiver-rated questionnaire developed to screen for BPSD in community and clinical settings; key behavioral issues such as aggression, agitation, and resistance to care are rated on a 5-point Likert scale ranging from 0 (never) to 4 (always). Moderate internal consistency of DBD-5 results was observed in this study (Cronbach α=0.67).

Daily social functioning was measured using the family-rated version of the Japanese Social Functioning in Dementia scale (SF-DEM-J) [30]. We selected its “Spending Time with Others” subscale for the purpose of this study because it captures clearly differentiated types of social activities that are relevant to those potentially supported or facilitated by smartphone functions [31]. This subscale contains 7 items related to social activities with friends or family, which broadly correspond to levels 3 and 4 of Levasseur taxonomy of social activities, encompassing social contact without performing a specific activity and performing an activity with others [32]. We opted for a carer-reported tool, as it has demonstrated higher reliability than patient-reported measures have [33]. Family members rated the social activities of the individuals with dementia over the previous month on a 4-point scale, ranging from 0 (never) to 3 (very often), with acceptable internal consistency (Cronbach α=0.69). Additionally, one question was derived from a different subscale of the SF-DEM-J to confirm that mobile phone use was associated with communication through phones.

#### Doctor’s Data Sheet

The doctors recorded information on participants’ dementia etiologies by selecting the following from a predefined list: Alzheimer disease, Lewy body dementia, frontotemporal dementia, vascular dementia, mixed dementia, others, or unknown. They administered the MMSE in accordance with standardized guidance and documented the resulting scores. Participants’ comorbidities were selected from a predefined list on the data collection form, and the total number of comorbidities was calculated for each participant.

### Data Analysis

The primary outcome of this study was the frequency of social activities, assessed using the “Spending Time with Others” subscale of the SF-DEM-J. The main exposure variable was the type of mobile phone used. Based on their self-reported usage, the participants were categorized into 3 groups, including smartphone users, cellphone users, and nonusers, with “use” defined as operating the device at least once a week. While the original study objective was to examine the association between smartphone use and social activity among individuals with dementia, a substantial portion of the participants reported using cellphones. Including the data of this group in the analysis provided an opportunity to explore a more nuanced gradient of technology use and its potential relationship with social engagement. Nonusers were used as the reference group in all analyses.

For objective (1), frequencies and percentages were calculated for each group (smartphone users, cellphone users, and nonusers) and compared using chi-square tests. Factors associated with the use of each device were modeled using multinomial logistic regression, with nonusers as the reference category. While exploring the most appropriate model using a list of candidate variables, we first eliminated those with weak associations (*P*<.20) in the chi-square tests. Spearman correlation coefficients were subsequently calculated among the remaining candidate variables. For each pair of variables with a correlation coefficient >0.40, one was considered for elimination. The variables retained in the final model were selected based on logical considerations and prior research evidence [34].

For objective (2), we assessed the descriptive data of 25 general smartphone apps and functions used by smartphone users.

For objective (3), we compared the total score for the frequency of social activities among smartphone or cellphone users with that among nonusers using linear regression analysis. To confirm the robustness of the association, comparisons were made using 3 different models: Crude, Model 1 that was adjusted for selected baseline characteristics, and Model 2 that was additionally adjusted for cognitive function. An item-level analysis of the frequency of each social activity was performed with adjustment for the Model 2 variables.

Missing values in the family-reported scales (ie, DASC-8, DBD-5, and SF-DEM-J subscale) accounted for less than 2% of the responses for each item; hence, they were imputed using the mean value of the family’s available responses on the respective scale. Statistical significance was set at *P*<.05, and the Bonferroni method was applied to adjust for multiple comparisons in the interpretation of *P* values in the pairwise comparisons. All statistical analyses were performed using R version 4.4.3 (R Foundation for Statistical Computing).

### Ethical Considerations

Written informed consent was obtained from the individuals with dementia and their family members to ensure that all parties were fully informed about the study’s purpose, procedures, potential risks, and benefits. They were also assured that their participation was entirely voluntary and that they could withdraw at any time without consequences. Their survey responses would remain confidential and neither be shared with their attending physicians nor linked to any clinical evaluations. In addition, data were anonymized and stored securely, with access limited to the research team. Participants received a small token of appreciation for their participation. All the study procedures were approved by the Tokyo Metropolitan Institute for Geriatrics and Gerontology Research Ethics Committee (approval number: R23-090).

## Results

### Baseline Characteristics

A total of 165 individuals with dementia were screened, among whom 151 participated in the survey (response rate 92%). Their mean age was 82.9 years (SD 6.6), 95 out of 151 (63%) participants were women, and their average MMSE score was 20.9 (SD 4.6). Dementia etiologies were Alzheimer disease (107/151, 71%), Lewy body dementia (17/151, 11%), mixed dementia (17/151, 11%), vascular dementia (3/151, 2%), frontotemporal dementia (1/151, 1%), and other or unknown (6/151, 4%). Out of 151 individuals, 43 were smartphone users (29%), 30 were cellphone users (20%), and 78 were nonusers (52%). The family members had a mean age of 63.9 years (SD 12.8), and out of 151 individuals, 112 (75%) were women. Their relationships with the participants were spouse (53/151, 35%), child (90/151, 60%), son- or daughter-in-law (4/151, 3%), and others (4/151, 3%). The majority (103/151, 68%) lived with the participants, and they spent time together on a daily (112/151, 74%), weekly (27/151, 18%), or monthly (12/151, 8%) basis.

Differences among participants recruited from hospitals and clinics were observed in several socioeconomic and functional characteristics (Multimedia Appendix 2). Participants recruited from the 4 hospitals tended to be younger, more often male, had higher educational attainment, were married, and reported a better perceived economic status than those recruited from the 13 primary care clinics. Participants recruited from hospitals also demonstrated slightly higher levels of cognitive and physical functioning.

### Mobile Phone Users

Smartphone users were significantly younger (*P*=.002), had more years of education (*P*=.002), and demonstrated better cognitive function (*P*=.004 for MMSE score and *P*<.001 for DASC-8 score) than nonusers did (Table 1). The multivariable predictive model (Table 2) confirmed that an older age was associated with a lower likelihood of smartphone use (odds ratio [OR] 0.41, 95% CI 0.21-0.80; *P*=.009), while more years of education (OR 1.82, 95% CI 1.00-3.30; *P*=.05) and a higher MMSE score (OR 2.14, 95% CI 1.24-3.69; *P*=.007) were positively associated with smartphone use. In addition, living alone was significantly associated with a higher likelihood of smartphone use (OR 3.17, 95% CI 1.08-9.31; *P*=.04).

**Table 1.** Participant characteristics by mobile phone use category.

Participant characteristics	Smartphone use (n=43 28.5%), n (%)		Cellphone use (n=30, 19.9%), n (%)		Nonuse (n=78, 51.7%), n (%)		Overall, *P* value	1 versus 3^a^, *P* value	2 versus 3, *P* value
Age (years)							.01	.002	.45
≤74	8 (38.1)		5 (23.8)		8 (38.1)				
75‐84	28 (37.8)		14 (18.9)		32 (43.2)				
≥85	7 (12.5)		11 (19.6)		38 (67.9)				
Sex							.07	.04	.07
Men	20 (35.7)		14 (25.0)		22 (39.3)				
Women	23 (24.2)		16 (16.8)		56 (58.9)				
Education (years)							.01	.002	.45
≤9	5 (11.9)		8 (19.0)		29 (69.0)				
10‐12	15 (28.3)		11 (20.8)		27 (50.9)				
≥13	23 (42.6)		11 (20.4)		20 (37.0)				
Marital status							.14	.04	.50
Married	28 (35.0)		16 (20.0)		36 (45.0)				
Single, divorced, or widowed	15 (21.1)		14 (19.7)		42 (59.2)				
Living arrangement							.18	.17	.09
Cohabiting	32 (26.9)		21 (17.6)		66 (55.5)				
Living alone	11 (34.4)		9 (28.1)		12 (37.5)				
Perceived economic status							.16	.04	.77
Good	21 (41.2)		10 (19.6)		20 (39.2)				
Fair	20 (23.5)		17 (20.0)		48 (56.5)				
Poor	2 (15.4)		3 (23.1)		8 (61.5)				
Number of comorbidities							.23	.39	.26
0	5 (26.3)		6 (31.6)		8 (42.1)				
1‐2	22 (36.1)		8 (13.1)		31 (50.8)				
≥3	16 (22.5)		16 (22.5)		39 (54.9)				
Dementia etiologies							.50	.56	.35
Alzheimer disease	28 (26.2)		25 (23.4)		54 (50.5)				
Lewy body dementia	5 (29.4)		1 (5.9)		11 (64.7)				
Mixed dementia	6 (35.3)		4 (23.5)		7 (41.2)				
Vascular dementia	0 (0.0)		0 (0.0)		3 (100.0)				
Frontotemporal dementia	1 (100.0)		0 (0.0)		0 (0.0)				
Others or unknown	3 (50.0)		0 (0.0)		3 (50.0)				
MMSE^b^ score							.015	.004	.12
≤19	9 (17.0)		8 (15.1)		36 (67.9)				
20‐23	15 (28.8)		12 (23.1)		25 (48.1)				
24‐30	19 (43.2)		10 (22.7)		15 (34.1)				
DASC-8^c^ score							<.001	<.001	.006
Intact cognitive function (≤10)	2 (22.2)		5 (55.6)		2 (22.2)				
Mild dementia (11-16)	24 (53.3)		8 (17.8)		13 (28.9)				
Mild to moderate dementia (≥17)	17 (17.7)		16 (16.7)		63 (65.6)				
DBD-5^d^ score							.16	.10	.19
5‐9	21 (36.8)		13 (22.8)		23 (40.4)				
10‐12	11 (21.6)		11 (21.6)		29 (56.9)				
13‐25	11 (26.2)		5 (11.9)		26 (61.9)				

a Differences across groups tested using chi-square test.

b MMSE: Mini-Mental State Examination.

c DASC-8: Dementia Assessment Sheet for Community-based Integrated Care System 8-item.

d DBD-5: Dementia Behavior Disturbance scale.

**Table 2.** Factors associated with mobile phone use compared to nonuse.

Participant factor^a^	Smartphone use				Cellphone use			
	OR^b^ (95% CI)			*P* value	OR (95% CI)			*P* value
Age (years), ≤74, 75-84, and ≥85 (years)	0.41 (0.21-0.80)			.009	0.67 (0.33-1.36)			.26
Gender (men and women)	0.68 (0.26-1.74)			.42	0.43 (0.16-1.17)			.10
Education (years), ≤9, 10-12, and ≥13 (years)	1.82 (1.00-3.30)			.05	1.08 (0.59-1.98)			.80
Living arrangement (cohabiting, living alone)	3.17 (1.08-9.31)			.04	3.37 (1.12-10.13)			.03
MMSE^c^ score (8-19, 20-23, 24-30)	2.14 (1.24-3.69)			.007	1.67 (0.94-2.98)			.08

a Multinomial logistic regression was conducted with nonusers as the reference category. All independent variables were categorized as shown in the parentheses, with the first category used as the reference group in the regression analysis.

b OR: odds ratio.

c MMSE: Mini-Mental State Examination.

Cellphone users presented less distinctive characteristics. Age, years of education, and MMSE score were not significantly associated with cellphone use (*P*=.45, *P*=.45, and *P*=.12, respectively; Table 1). In the multivariable predictive model, women were marginally less likely to use cellphones (OR 0.43, 95% CI 0.16-1.17; *P*=.10), and a higher MMSE score was marginally associated with cellphone use (OR 1.67, 95% CI 0.94-2.98; *P*=.08). The only factor that was significantly associated with cellphone use was living arrangements: cellphone users were 3.37 times more likely to live alone (OR 3.37, 95% CI 1.12-10.13; *P*=.03).

### Patterns of Smartphone Use

Among the 43 smartphone users, more than 3-quarters had been using a smartphone for longer than 3 years. Specifically, the duration of smartphone use was up to 1 year for 5 out of 43 (11%) individuals, up to 3 years for 5 out of 43 (11%) individuals, up to 5 years for 14 out of 43 (32%) individuals, and up to 10 years for 20 out of 43 (46%) individuals.

**Figure 1.**
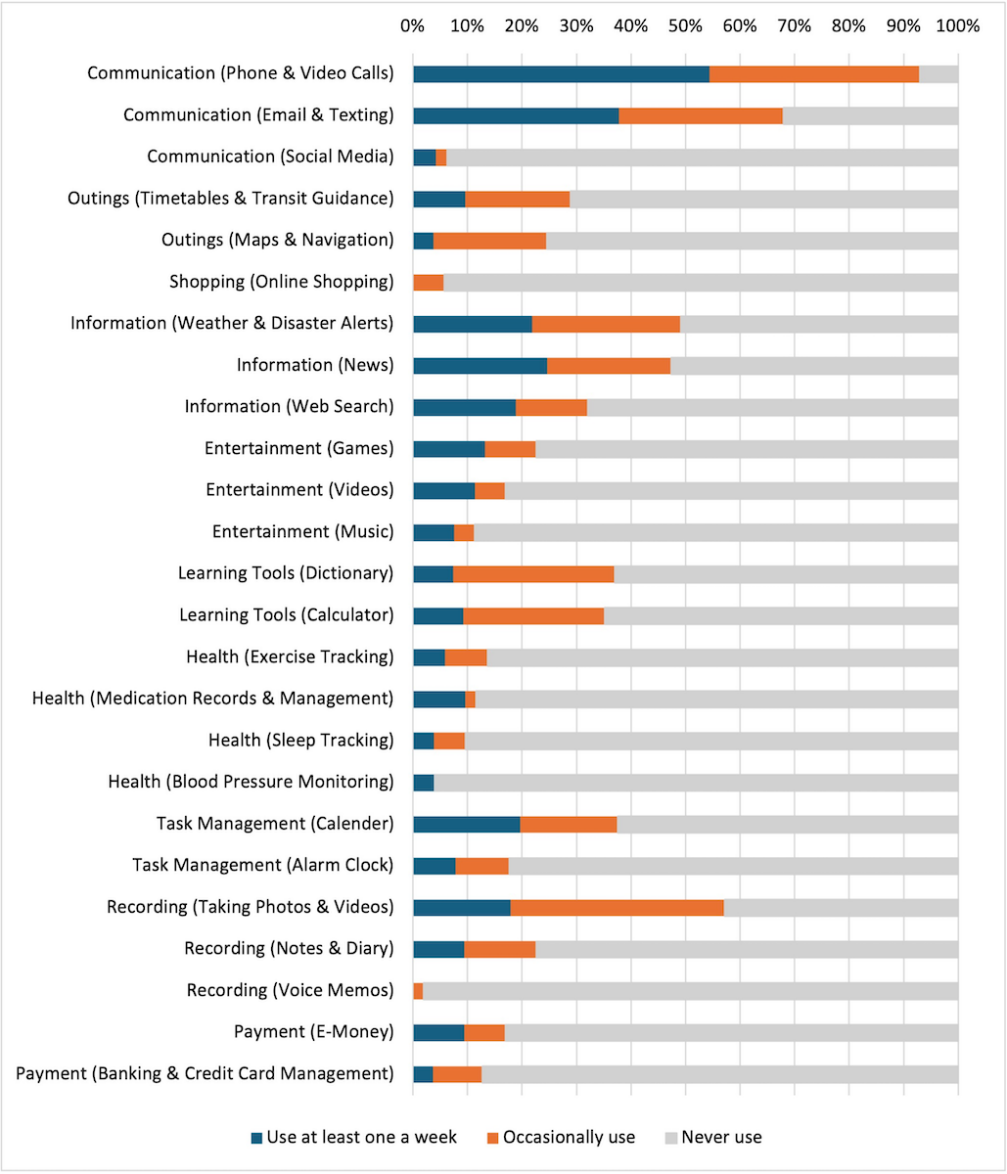
Purposes of smartphone use (n=43).

Smartphones were used most often for communication, reading the news, and taking photos and videos (Figure 1). Out of 43 smartphone users, 40 (93%) made phone and video calls more than once a week or occasionally, while 27 (63%) used email and texting. Approximately half of the smartphone users used weather and disaster alerts (20/43, 47%), accessed news (20/43, 47%), and took photos and videos (24/43, 56%). However, apps and functions that could assist out-of-home activities were less likely to be used, as only 11 out of 40 (26%) used transit guidance and navigation systems and 7 out of 40 (16%) made digital payments. Regarding cognitive aid features, 16 out of 40 (37%) used calendars, 9 out of 40 (21%) used notes and diaries, and 7 out of 40 (16%) used alarm clocks. Health monitoring or tracking apps (such as for exercise, medication, and sleep) were used by 5 out of 40 (12%) or fewer smartphone users.

### Mobile Phone Use and Participation in Social Activities

The mean scores on the “Spending Time with Others” subscale of the SF-DEM-J were 6.0 (SD 3.6) for smartphone users, 5.1 (SD 4.1) for cellphone users, and 4.4 (SD 3.2) for nonusers. We first confirmed that mobile phone use was associated with more frequent telephonic communication with friends and family members. After adjusting for age, sex, years of education, living arrangements, and MMSE score, both smartphone use (b=1.07, 95% CI 0.73-1.41; *P*<.001) and cellphone use (b=0.73, 95% CI 0.37-1.10; *P*=.001) showed significant associations.

Table 3 shows the results of the stepwise linear regression analyses examining the association between mobile phone use and the frequency of social activities, as measured using the “Spending Time with Others” subscale of the SF-DEM-J, with nonusers as the reference group. In the crude model, smartphone use was significantly associated with higher social activity scores (b=1.55, 95% CI 0.24-2.86; *P*=.02). This association remained marginally significant after adjusting for age, sex, years of education, and living arrangements in Model 1 (b=1.26, 95% CI −0.13 to 2.64; *P*=.07) and additionally for MMSE score in Model 2 (b=1.41, 95% CI −0.01 to 2.83; *P*=.05). Cellphone use, by contrast, was not significantly associated with social activity levels in any of the models.

**Table 3.** Association between mobile phone use and frequency of social activities.

Participant factor	Crude^a^				Model 1^b^				Model 2^c^			
	b (95% CI)			*P* value	b (95% CI)			*P* value	b (95% CI)			*P* value
Smartphone use	1.55 (0.24 to 2.86)			.02	1.26 (–0.13 to 2.64)			.07	1.41 (–0.01 to 2.83)			.05
Cellphone use	.60 (–0.90 to 2.09)			.44	.58 (–0.92 to 2.09)			.45	.68 (–0.83 to 2.20)			.38
Age (years), 65-74, 75-84, and ≥85 (years)	–1.13 (–1.96 to −0.30)			.008	–.87 (–1.75 to 0.02)			.06	–.80 (–1.70 to 0.09)			.08
Gender (men and women)	–.19 (–1.38 to 1.01)			.76	.62 (–0.67 to 1.91)			.35	.56 (–0.73 to 1.86)			.39
Education (years) ≤9, 10-12, and ≥13 (years)	.59 (–0.13 to 1.30)			.11	.27 (–0.51 to 1.04)			.50	.33 (–0.46 to 1.11)			.41
Living arrangement (cohabiting or living alone)	–.68 (–2.08 to 0.73)			.35	–.77 (–2.22 to 0.69)			.30	–.76 (–2.21 to 0.69)			.30
MMSE^d^ score (8-19, 20-23, and 24-30)	–.13 (–0.84 to 0.58)			.72	—^e^			—	–.34 (–1.07 to 0.39)			.36

a Stepwise linear regression analysis was conducted to assess the association between mobile phone use and the subscale score, with nonusers as the reference group.

b Model 1 adjusted for baseline characteristics (age, sex, education, and living arrangement).

c Model 2 additionally adjusted for cognitive function (MMSE).

d MMSE: Mini-Mental State Examination.

e Not applicable.

**Figure 2.**
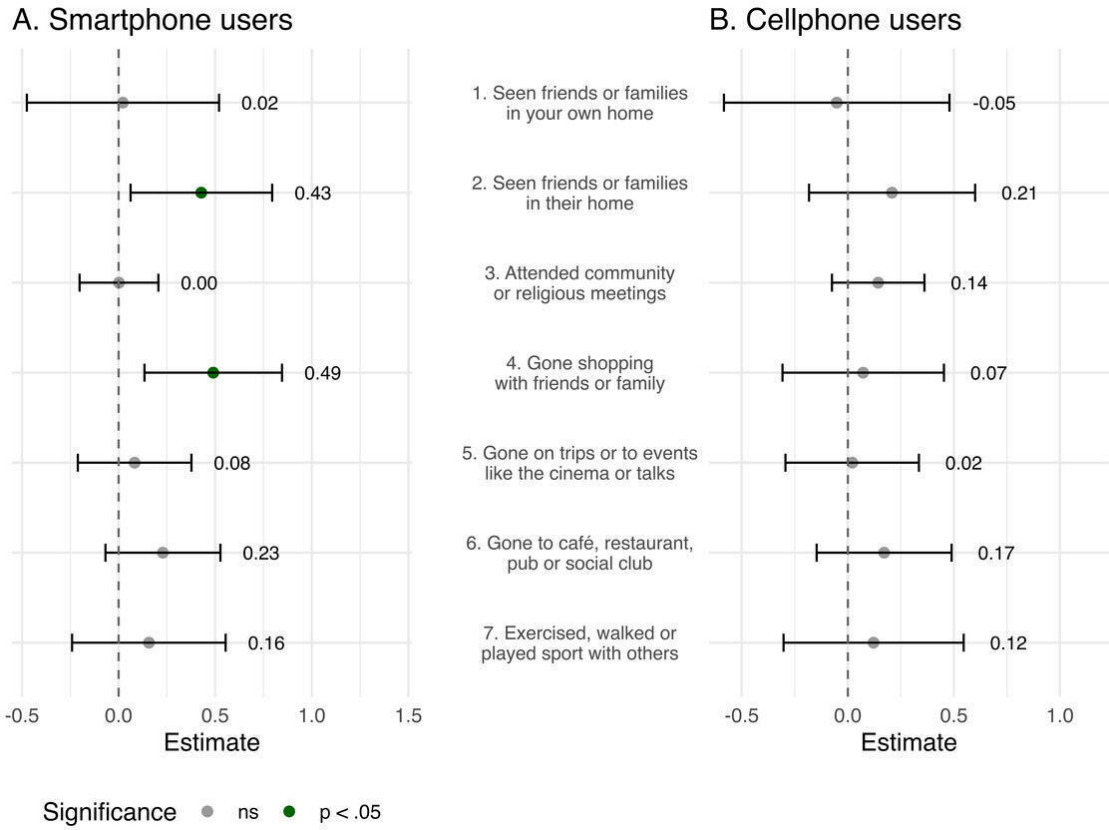
Forest plot of item-level associations.

Figure 2 presents the item-level analysis of the association between mobile phone use and the frequency of specific social activities, adjusted for the covariates in Model 2. Smartphone users reported significantly more frequent visits to the homes of friends or family members (b=0.43, 95% CI 0.08-0.78; *P*=.02) and shopping trips with friends or family members (b=0.49, 95% CI 0.15-0.83; *P*=.005) than did nonusers. Most of the other social activities showed nonsignificant but positive associations with smartphone use. In contrast, among cellphone users, none of the individual items reached statistical significance, although many showed positive trends.

All estimates were adjusted for baseline characteristics and cognitive function, including age, sex, education, living arrangements, and MMSE scores.

## Discussion

### Principal Findings

In this cross-sectional study of community-dwelling older adults with mild to moderate dementia, 29% were regular smartphone users, most for longer than 3 years. Smartphone use was independently associated with a younger age, more years of education, living alone, and better cognitive function. The top five smartphone uses were (1) making phone and video calls, (2) sending emails and texts, (3) taking photos and videos, (4) checking the weather, and (5) reading the news. Functions that could more directly aid cognitive function (eg, notes and alarms) and out-of-home social participation (eg, GPS navigation and digital payments) were not often used. Although both smartphone and cellphone users reported more frequent telephonic communication with their friends or family members, only smartphone use was associated with a higher overall score for participation in social activities after adjusting for demographic and cognitive variables. At the activity level, smartphone users reported significantly more frequent visits to the homes of friends and family members and more frequent shopping trips with friends and family members than did nonusers.

### Predictors of Smartphone Use

While our participants used smartphones less often than the general older adult population does, their adoption was observed among a substantial proportion of older adults with dementia in their 70s and 80s (mean age 82.9, SD 6.6 years). In 2023, the rates of smartphone use among the general older adult population in Japan were 71% for those aged 65‐74 years and 38% for those aged 75 years and older [17]. Among our participants with dementia in the same age groups, the rates were lower at 38% and 23%, respectively. Directly comparable data are limited because most studies on smartphone use among individuals with dementia are feasibility or qualitative studies with small sample sizes [10,20,35]; nevertheless, these studies provide some context. In 2016, 75% of 53 individuals (mean age 64.9, SD 13.3 years) who visited a memory clinic for cognitive evaluation in the United States owned a smartphone [36]. In France (2013‐2015), 33% of 323 older adults (mean age 75.9, SD 7.0 years) who underwent cognitive assessment at a memory clinic reported the regular use of smartphones or tablets [18]. Considering that these rates are from approximately a decade ago, when smartphone adoption among the general older population was 25%‐55% lower [16], the relatively low use among our participants likely reflects their older age and lower cognitive function rather than a persistent trend among individuals with dementia. As smartphone use has become more widespread among older adults in general [16,17], a similar increase can be expected in individuals with dementia.

Consistent with previous findings, we found that a younger age, higher educational level, and better cognitive function were associated with smartphone use [10]. Notably, living alone emerged as a significant predictor of smartphone use. This trend has rarely been explored [10] but may reflect the increasing number of individuals with dementia who live alone [37] and the heightened risk of loneliness in this group [38]. Smartphones may serve as a compensatory tool for maintaining independent living and social connections among these individuals, and this warrants further investigation [39].

### Usage Patterns

Commonly reported smartphone uses included communication, searching for information, and taking photos or videos, while the use of features that could more specifically aid cognitive function and out-of-home activities was limited. Similar trends were reported in a US study conducted in 2016, where social and general-purpose functions (eg, email, photos, and news) were favored over cognitive aids (directions, to-do lists, alarms, and reminders) [36]. However, a more recent UK study (2021), in which 29 younger adults with dementia (aged 56‐78 years) were interviewed, revealed that they actively explored and enjoyed using various smartphone features, including calendars, reminders, and health-tracking apps [20]. These individuals likely developed smartphone proficiency before cognitive decline, which enabled them to integrate EICTs into their daily lives despite their impairments [39]. Given that cognitive aid features have proven both preferred and effective in prior research [10,14], our findings suggest that older individuals such as those who participated in our study may benefit from additional support to effectively use them [34].

### Associations With Participation in Social Activities

Although both smartphone and cellphone use were associated with more frequent phone communication with friends and family members, only smartphone use was associated with greater overall social activity. Prior research has revealed that research-based assistive technologies and EICTs, which typically apply cognitive aid features and are tailored to the needs of individuals with dementia, can supplement daily cognitive functions [10,12] and enhance social participation [11]. Our findings suggest that the benefits of these specialized technologies may also be transferable to commercially available smartphones. However, given the limited usage patterns observed among our participants, there remains untapped potential to expand the impact of smartphones through improved design and tailored support [14,20].

Smartphone use was significantly associated with visiting the homes of friends and family members and shopping together, with a marginal significance also observed for going to cafés, restaurants, pubs, or social clubs. In contrast, no significant association was found for attending community or religious meetings, going on trips, or participating in events. These findings align with those of a multinational study of 163 individuals with mild dementia, in which the authors reported that out-of-home participation was most frequent in familiar, informal activities such as visiting friends and local shopping, whereas engagement in more complex or unfamiliar activities was less common [40]. This pattern suggests that individuals with dementia use smartphones to stay engaged in familiar, emotionally rewarding routines that require minimal cognitive planning [8]. Accordingly, support strategies should be focused on enabling them to sustain meaningful activities with familiar social connections rather than on expanding participation into less familiar domains.

### Implications

Our findings suggest that commercially available smartphones can serve as meaningful tools for older adults with dementia to maintain social connections and engage in valued social activities. However, the predominant use of basic communication and information functions indicates a missed opportunity to leverage the full potential of smartphone features that can promote independence and social participation. Given the promise of EICT training interventions [21], tailored strategies are needed to help individuals with dementia to use smartphone functions in personally meaningful ways, focusing on preferred activities and offering training in practical tools such as calendars, reminders, and digital payment systems that can directly support these activities.

### Strengths and Limitations

This study has several strengths. First, multisite recruitment reflecting collaborative dementia care systems involving medical centers for dementia and primary care clinics enhanced sample diversity, which improved the robustness of the findings across different care settings. Participants recruited from hospitals and primary care clinics differed in sociodemographic and functional characteristics. Second, the inclusion of both patient- and caregiver-reported data allowed for a more comprehensive understanding of smartphone use and social participation, particularly in cases where cognitive limitations might have affected self-reporting. Third, the item-level analysis of social activities provided more nuanced insights into how smartphone use relates to specific aspects of everyday life.

However, several limitations should also be noted. The cross-sectional design prevents any conclusions about causality between smartphone use and social activity. Additionally, reliance on caregiver-reported outcomes may introduce reporting bias, particularly in cases where caregivers had limited knowledge of the participants’ daily routines. Finally, the findings may not be generalizable to individuals with more advanced dementia or those not receiving regular medical care, as this study focused on community-dwelling older adults with mild-to-moderate dementia who were recruited through outpatient dementia services.

### Future Research Directions

In the future, researchers should examine how spontaneous smartphone use changes over the course of cognitive decline and whether sustained use contributes to better social and psychosocial outcomes. Longitudinal studies are required to clarify these relationships. Moreover, efforts should be focused on identifying effective ways to support and expand the use of commercially available smartphones among older adults with dementia, particularly to enhance their participation in meaningful social activities. Given the limited use of cognitive aid and out-of-home activity features observed in our study, targeted training or supportive environments would reduce barriers and facilitate more effective use of these functions. Collaborating with individuals with dementia in the design of such training programs and environments may further enhance their relevance and effectiveness.

### Conclusions

Our findings highlight that a substantial proportion of community-dwelling older adults with mild to moderate dementia continue to use smartphones primarily for basic communication and information access. While smartphone use was associated with greater telephonic contact and overall social activity, apps and functions that could support independence and social participation were underused. These findings emphasize the potential of commercially available smartphones as meaningful tools for maintaining social engagement in dementia care. Targeted support may help bridge the gap between current usage and the broader capabilities of these devices, enabling individuals with dementia to sustain valued routines and social participation in daily life.

## Supplementary material

10.2196/81927 Multimedia Appendix 1 Survey items on smartphone apps and functions.

10.2196/81927 Multimedia Appendix 2 Characteristics of participants recruited from hospitals and primary care clinics.

## Abbreviations

BPSD: Behavioral and psychological symptoms of dementia
DASC-8: Dementia Assessment Sheet for Community-based Integrated Care System 8-items
DBD-5: Dementia Behavior Disturbance scale
EICT: Everyday Information and Communication Technology
MMSE: Mini-Mental State Examination
OR: odds ratio
SF-DEM-J: Japanese Social Functioning in Dementia scale

## References

[1] Nichols E, Steinmetz JD, Vollset SE (2022). Estimation of the global prevalence of dementia in 2019 and forecasted prevalence in 2050: an analysis for the Global Burden of Disease Study 2019. Lancet Public Health.

[2] Gauthier S, Webster C, Servaes S, Morais JA, Rosa-Neto P (2022). World Alzheimer report 2022: life after diagnosis: navigating treatment, care and support. https://www.alzint.org/u/World-Alzheimer-Report-2022.pdf.

[3] Clare L, Gamble LD, Martyr A (2022). Longitudinal trajectories of quality of life among people with mild-to-moderate dementia: a latent growth model approach with IDEAL cohort study data. J Gerontol B Psychol Sci Soc Sci.

[4] Wolverson EL, Clarke C, Moniz-Cook ED (2016). Living positively with dementia: a systematic review and synthesis of the qualitative literature. Aging Ment Health.

[5] Martyr A, Nelis SM, Quinn C (2018). Living well with dementia: a systematic review and correlational meta-analysis of factors associated with quality of life, well-being and life satisfaction in people with dementia. Psychol Med.

[6] Dröes RM, Chattat R, Diaz A (2017). Social health and dementia: a European consensus on the operationalization of the concept and directions for research and practice. Aging Ment Health.

[7] Hackett RA, Steptoe A, Cadar D, Fancourt D (2019). Social engagement before and after dementia diagnosis in the English longitudinal study of ageing. PLoS ONE.

[8] Bohn L, Kwong See ST, Fung HH (2019). Preference for emotionally meaningful activity in Alzheimer’s disease. Aging Ment Health.

[9] Evans J, Brown M, Coughlan T, Lawson G, Craven MP, Kurosu M (2015). Human-Computer Interaction: Interaction Technologies.

[10] Wilson SA, Byrne P, Rodgers SE, Maden M (2022). A systematic review of smartphone and tablet use by older adults with and without cognitive impairment. Innov Aging.

[11] Schepens Niemiec SL, Lee E, Saunders R, Wagas R, Wu S (2023). Technology for activity participation in older people with mild cognitive impairment or dementia: expert perspectives and a scoping review. Disabil Rehabil Assist Technol.

[12] Lancioni G, Desideri L, Singh N, O’Reilly M, Sigafoos J (2021). Technology options to help people with dementia or acquired cognitive impairment perform multistep daily tasks: a scoping review. JET.

[13] Van der Roest HG, Wenborn J, Pastink C, Dröes RM, Orrell M (2017). Assistive technology for memory support in dementia. Cochrane Database Syst Rev.

[14] Thorpe JR, Rønn-Andersen KVH, Bień P, Özkil AG, Forchhammer BH, Maier AM (2016). Pervasive assistive technology for people with dementia: a UCD case. Healthc Technol Lett.

[15] Ye B, Chu CH, Bayat S, Babineau J, How TV, Mihailidis A (2023). Researched apps used in dementia care for people living with dementia and their informal caregivers: systematic review on app features, security, and usability. J Med Internet Res.

[16] Pew Research Center (2022). 3 internet, smartphone, and social media use. https://www.pewresearch.org/wp-content/uploads/sites/20/2022/12/PG_2022.12.06_Online-Civic-Engagement_REPORT.pdf.

[17] (2023). Public opinion poll on the use of telecommunications equipment. The Cabinet Office.

[18] Wu YH, Lewis M, Rigaud AS (2019). Cognitive function and digital device use in older adults attending a memory clinic. Gerontol Geriatr Med.

[19] Wallcook S, Nygård L, Kottorp A, Malinowsky C (2021). The use of everyday information communication technologies in the lives of older adults living with and without dementia in Sweden. Assist Technol.

[20] Wilson SA, Byrne P, Rodgers SE (2024). “I’d be lost without my smartphone”: a qualitative analysis of the use of smartphones and tablets by people living with dementia, mild cognitive impairment, and their caregivers. Aging Ment Health.

[21] Kerkhof YJF, Bergsma A, Mangiaracina F, Planting CHM, Graff MJL, Dröes RM (2022). Are people with mild dementia able to (re)learn how to use technology? A literature review. Int Psychogeriatr.

[22] Kwan RYC, Cheung DSK, Kor PPK (2020). The use of smartphones for wayfinding by people with mild dementia. Dementia (London).

[23] (2004). Ten-year plan to understand dementia and build community networks. Ministry of Health, Labour and Welfare.

[24] Ministry of Health, Labour and Welfare (2024). Basic plan for the promotion of policies on dementia. https://www.mhlw.go.jp/content/12300000/001347978.pdf.

[25] Reisberg B (1988). Functional assessment staging (FAST). Psychopharmacol Bull.

[26] (2024). White paper information and communications in japan. https://www.soumu.go.jp/johotsusintokei/whitepaper/eng/WP2024/2024-index.html.

[27] Neal DP, Kuiper L, Pistone D (2023). FindMyApps eHealth intervention improves quality, not quantity, of home tablet use by people with dementia. Front Med (Lausanne).

[28] Toyoshima K, Araki A, Tamura Y (2018). Development of the dementia assessment sheet for community-based integrated care system 8-items, a short version of the dementia assessment sheet for community-based integrated care system 21-items, for the assessment of cognitive and daily functions. Geriatr Gerontol Int.

[29] Ito K, Ogisawa F, Furuta K, Awata S, Toba K (2021). Development of a five-item short-form version of the Dementia Behavior Disturbance Scale. Geriatr Gerontol Int.

[30] Umeda S, Kanemoto H, Suzuki M (2024). Validation of the Japanese version of the Social Functioning in Dementia scale and COVID-19 pandemic’s impact on social function in mild cognitive impairment and mild dementia. Int Psychogeriatr.

[31] Budgett J, Brown A, Daley S (2019). The social functioning in dementia scale (SF-DEM): exploratory factor analysis and psychometric properties in mild, moderate, and severe dementia. Alzheimers Dement (Amst).

[32] Levasseur M, Richard L, Gauvin L, Raymond E (2010). Inventory and analysis of definitions of social participation found in the aging literature: proposed taxonomy of social activities. Soc Sci Med.

[33] Sommerlad A, Singleton D, Jones R, Banerjee S, Livingston G (2017). Development of an instrument to assess social functioning in dementia: the Social Functioning in Dementia scale (SF-DEM). Alzheimers Dement (Amst).

[34] Conway A, Ryan A, Harkin D, Mc Cauley C, Goode D (2023). A review of the factors influencing adoption of digital health applications for people living with dementia. Digit Health.

[35] Dixon E, Michaels R, Xiao X (2022). Mobile phone use by people with mild to moderate dementia: uncovering challenges and identifying opportunities: mobile phone use by people with mild to moderate dementia. ASSETS.

[36] Benge JF, Dinh KL, Logue E, Phenis R, Dasse MN, Scullin MK (2020). The smartphone in the memory clinic: a study of patient and care partner’s utilisation habits. Neuropsychol Rehabil.

[37] Portacolone E, Cohen AB (2024). Living alone with dementia: a reality check. Am J Geriatr Psychiatry.

[38] Clare L, Martyr A, Henderson C (2020). Living alone with mild-to-moderate dementia: findings from the IDEAL cohort. J Alzheimers Dis.

[39] Talbot CV, Briggs P (2022). The use of digital technologies by people with mild-to-moderate dementia during the COVID-19 pandemic: a positive technology perspective. Dementia (London).

[40] Thalén L, Malinowsky C, Margot-Cattin I (2022). Out-of-home participation among people living with dementia: a study in four countries. Dementia (London).

