# Supplementary material for: Smartphone Use and Social Activities Among People With Mild to Moderate Dementia: Multi-Informant Cross-Sectional Study
Source: JMIR Aging. 2026 Apr 24;9:e81927. doi: 10.2196/81927 (PMC13108958; doi:10.2196/81927)
Supplement: Multimedia Appendix 1 [file aging-v9-e81927-s001.docx]

Survey items on smartphone applications and functions

Which smartphone applications and functions do you use? How often do you use them?

(Answer this section only if you use a smartphone.)

| Purpose | Application/Function | At least once a week | Occasionally | Never |
| --- | --- | --- | --- | --- |
| Communication | Phone, video calls | 1 | 2 | 3 |
|  | Email, SNS (LINE etc.) | 1 | 2 | 3 |
|  | Social media (Twitter etc.) | 1 | 2 | 3 |
| Outings | Time tables, transit guidance | 1 | 2 | 3 |
|  | Maps, Navigation | 1 | 2 | 3 |
| Shopping | Online shopping | 1 | 2 | 3 |
| Information | Weather, disaster alerts | 1 | 2 | 3 |
|  | News | 1 | 2 | 3 |
|  | Web search | 1 | 2 | 3 |
| Entertainment | Games | 1 | 2 | 3 |
|  | Videos | 1 | 2 | 3 |
|  | Music, pod cast | 1 | 2 | 3 |
| Learning tools | Dictionary | 1 | 2 | 3 |
|  | Calculator | 1 | 2 | 3 |
| Health | Sleep tracking | 1 | 2 | 3 |
|  | Exercise tracking | 1 | 2 | 3 |
|  | Blood pressure monitoring | 1 | 2 | 3 |
|  | Medication records, medication management | 1 | 2 | 3 |
| Task management | Calendar | 1 | 2 | 3 |
|  | Alarm clock | 1 | 2 | 3 |
| Recording | Notes, diary | 1 | 2 | 3 |
|  | Taking photos and videos | 1 | 2 | 3 |
|  | Voice memos | 1 | 2 | 3 |
| Payment | E-money, QR code payments, Transportation IC cards | 1 | 2 | 3 |
|  | Online banking, Credit card management | 1 | 2 | 3 |

Characteristics of participants recruited from hospitals and primary care clinics

|  |  | Participants recruited from hospitals (n=49) | | Participants recruited from clinics (n=102) | |  |
| --- | --- | --- | --- | --- | --- | --- |
|  |  | n/mean | %/SD | n/mean | %/SD | p |
| Age | mean (SD) | 81.7 | 6.8 | 83.5 | 6.5 | 0.13 |
| Sex | Men | 23 | 46.9% | 33 | 32.4% | 0.082 |
|  | Women | 26 | 53.1% | 69 | 67.6% |  |
| Education | ≤9 years | 5 | 10.2% | 37 | 37.0% | <0.001 |
|  | 10–12 years | 18 | 36.7% | 35 | 35.0% |  |
|  | 13≤ years | 26 | 53.1% | 28 | 28.0% |  |
| Marital status | Married | 32 | 65.3% | 48 | 47.1% | 0.035 |
|  | Single/divorced/widowed | 17 | 34.7% | 54 | 52.9% |  |
| Living arrangement | cohabiting | 41 | 83.7% | 78 | 76.5% | 0.31 |
|  | Living alone | 8 | 16.3% | 24 | 23.5% |  |
| Perceived economic status | Good | 20 | 40.8% | 31 | 31.0% | 0.025 |
|  | Fair | 29 | 59.2% | 56 | 56.0% |  |
|  | Poor | 0 | 0.0% | 13 | 13.0% |  |
| Number of comorbidities | 0 | 6 | 12.2% | 13 | 12.7% | 0.19 |
|  | 1–2 | 15 | 30.6% | 46 | 45.1% |  |
|  | 3≤ | 28 | 57.1% | 43 | 42.2% |  |
| MMSE score | mean (SD) | 21.7 | 4.7 | 20.5 | 4.5 | 0.13 |
| DASC-8 score | mean (SD) | 16.7 | 4.2 | 18.4 | 4.5 | 0.034 |
| DBD-5 score | mean (SD) | 10.8 | 3.3 | 10.5 | 3.2 | 0.59 |

P-values were calculated using the chi-square test for categorical variables and t test for continuous variables.
